# The Role of Ivabradine in the Management of Angina Pectoris

**DOI:** 10.1007/s10557-016-6678-x

**Published:** 2016-07-30

**Authors:** Alessandra Giavarini, Ranil de Silva

**Affiliations:** 1 NIHR Biomedical Research Unit, Royal Brompton and Harefield NHS Foundation Trust, London, UK; 2 ICMS, Royal Brompton and Harefield NHS Foundation Trust, London, UK; 3 National Heart and Lung Institute (Brompton Campus), Imperial College London, Level 2 Chelsea Wing, London, SW3 6NP UK

**Keywords:** Ivabradine, I*f* current inhibitor, Angina pectoris, Stable coronary artery disease

## Abstract

Stable angina pectoris affects 2–4 % of the population in Western countries and entails an annual risk of death and nonfatal myocardial infarction of 1–2 % and 3 %, respectively. Heart rate (HR) is linearly related to myocardial oxygen consumption and coronary blood flow, both at rest and during stress. HR reduction is a key target for the prevention of ischemia/angina and is an important mechanism of action of drugs which are recommended as first line therapy for the treatment of angina in clinical guidelines. However, many patients are often unable to tolerate the doses of beta blocker or non-dihydropyridine calcium antagonists required to achieve the desired symptom control. The selective pacemaker current inhibitor ivabradine was developed as a drug for the management of patients with angina pectoris, through its ability to reduce HR specifically. The available data suggest that ivabradine is a well-tolerated and effective anti-anginal agent and it is recommended as a second-line agent for relief of angina in guidelines. However, recent clinical trials of ivabradine have failed to show prognostic benefit and have raised potential concerns about safety. This article will review the available evidence base for the current role of ivabradine in the management of patients with symptomatic angina pectoris in the context of stable coronary artery disease.

## Background

Coronary artery disease (CAD) is the global leading cause of mortality, accounting for approximately 7.4 million deaths annually. Furthermore, CAD accounts for a high morbidity rate, poor quality of life with a major socioeconomic impact. Stable angina pectoris affects 2–4 % of the population in Western countries, with prevalence increasing with age [1]. It entails an annual risk of death and nonfatal myocardial infarction (MI) of 1–2 % and 3 %, respectively [2, 3]. Optimal medical treatment (OMT), the cornerstone of stable CAD (SCAD) management, should be routinely offered to patients with SCAD to relieve angina and improve prognosis [3–5]. Guidelines recommend that OMT should comprise 2 anti-anginal drugs, one of which should be a beta blocker or calcium antagonist, in addition to disease modifying medications that are known to reduce risk of death and myocardial infarction (aspirin, statins and ACE inhibitors) [3–5]. Ivabradine is recommended as a second-line agent for relief of angina. This article will review the evidence base supporting the use of ivabradine in the management of patients with SCAD.

## Heart Rate as a Therapeutic Target in SCAD

Angina pectoris is principally caused by myocardial ischaemia, which arises from a mismatch between myocardial oxygen supply and demand. Relief of angina can be achieved pharmacologically by redressing this imbalance. Myocardial oxygen demand is mainly determined by the frequency and force of myocardial contraction. Heart rate (HR) is linearly related to myocardial oxygen consumption and coronary blood flow, both at rest and during stress. Furthermore, since myocardial perfusion occurs predominantly during diastole, there is an inverse relationship between the perfusion time and HR, with subendocardial perfusion being particularly sensitive to increased HR [6, 7].

Increased HR commonly precedes effort-induced ischemia and the frequency of ischaemic episodes is twice as high in patients with a resting HR >80 bpm as compared to those with HR <70 bpm. In addition, increased HR is an independent risk factor for ischaemia-triggered arrhythmia, infarct size and mortality [7]. Therefore, decreasing HR is a rational strategy to reduce myocardial ischaemia and prevent the development of symptomatic angina. In patients with previous MI, mortality was observed to increase when HR exceeded 60 bpm, [8] which has informed guideline recommendations to aim for a target HR of 55–60 bpm in patients with SCAD [3, 4]. Both beta blockers and non-dihydropyridine calcium antagonists reduce HR, with the degree of angina reduction being directly related to the magnitudes of HR reduction [8]. However, many patients cannot tolerate the doses of beta blocker required to achieve the desired level of HR reduction [9, 10] and non-DHP calcium antagonists should be avoided in patients with low blood pressure, left ventricular dysfunction or heart failure. Therefore drugs which selectively reduce HR and which are well tolerated would represent a biologically plausible and important therapeutic development for the management of patients with SCAD.

## Pharmacology and Anti-Ischaemic Action of Ivabradine

The “funny” current (I_*f*_) was identified within sinoatrial nodal cells and discovered to be primarily responsible for the spontaneous diastolic depolarisation that characterises pacemaker cells. The I_*f*_ current, also known as the “pacemaker current”, consists of mixed sodium–potassium ion influx which is activated during diastolic hyperpolarisation, thereby initiating diastolic depolarisation, the slope of which determines the sinoatrial node automaticity and hence sinus HR [11]. In addition to being located in the sinoatrial node, I_*f*_ channels are expressed throughout the cardiac conduction system, including the atrioventricular node and Purkinje fibres, where they are activated at more negative voltages. More recently, I_*f*_ channels have also been identified in the myocardial sleeves extending around the pulmonary veins [12, 13].

**Fig. 1:**
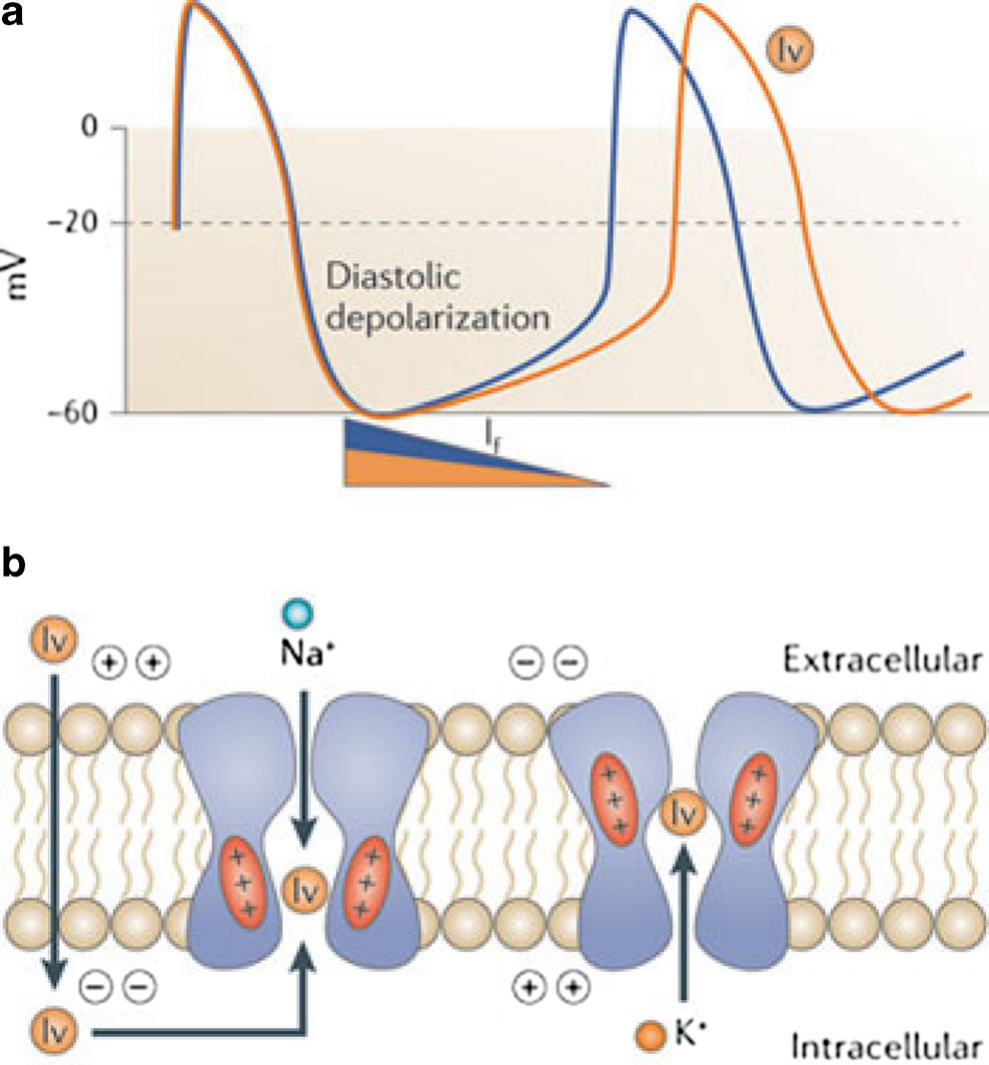
Mechanism of action of ivabradine on sinoatrial *I*_f_ current. **a** | Hyperpolarization-activated cyclic nucleotide-gated (HCN) channels allow the passage of the funny current (*I*_f_), which is a major current producing slow diastolic depolarization of the sinoatrial pacemaker potential. The other currents contributing to diastolic depolarization are omitted. Ivabradine (‘Iv’) blocks HCN channels and reduces the slope of diastolic depolarization, thereby reducing the frequency of sinoatrial pacemaker potentials. **b** | Current-dependent block of HCN channels by ivabradine. Ivabradine enters the pore of HCN channels from the intracellular aspect when the channels are in the open conformation (*left side of panel*). When the membrane potentials are more positive than ~ −20 mV, HCN-mediated currents are mainly carried by K^+^ and outwardly directed. The outward current drives ivabradine into the pore of the channel, where it binds tightly to a specific site (*right side of panel*). The polarity of the plasma membrane is indicated by ‘+’ or ‘−’ signs. The movement of the positively charged S4 helix of HCN channels in the electrical field is also indicated. Reproduced from [14]

Ivabradine is the only clinically approved I_*f*_ current inhibitor. It produces a dose-dependent, selective, inhibition of the I_*f*_ current by binding to the cation channel pore in its open conformation, thereby decreasing the slope of diastolic depolarisation, prolonging sinus nodal cell recovery time and decreasing HR (Fig. 1) [14, 15]. The magnitude of HR reduction is greatest in individuals with the highest resting HR [16, 17]. Ivabradine-induced HR reduction is not subject to tolerance and occurs at concentrations that do not interfere with other ionic channels. In contrast to beta blockers and non-DHP-calcium channel blockers, ivabradine-induced HR reduction at clinically approved doses does not affect myocardial contractility, atrioventricular conduction or ventricular repolarisation [1, 11]. Ivabradine induced heart rate reduction has been shown to improve diastolic dysfunction and ameliorate myocardial hypoxia in experimental models [15].

Mechanistic studies also suggest potential additional modes of benefit of ivabradine. In comparison with bisoprolol, ivabradine treatment was associated with increased maximal hyperaemic flow and coronary flow reserve, despite a similar reduction in HR [15]. This may be explained by increased diastolic perfusion time, enhanced isovolumic contraction and recruitment of collateral flow. Studies in a mouse model of hind limb ischaemia suggest that ivabradine promotes angiogenesis [18] and recent invasive assessments of collateral function in patients with SCAD suggest that HR reduction by ivabradine is associated with prolongation of diastolic perfusion and improved collateral function, potentially through altered tangential shear force at the endothelial surface or enhanced vascular stretch which may both promote arteriogenesis [19]. Ivabradine induced HR reduction may also have a direct anti-atherogenic effect and improve endothelial dysfunction [20, 21]. In a porcine model of acute ischaemia, ivabradine induced improvements in myocardial blood flow and wall thickening in the ischaemic zone could be reversed through atrial pacing, but the reduction in infarct size could not [22]. Inhibition of I*f* channels at the level of the ventricular myocyte may confer a direct cardioprotective effect through enhanced cardiac myocyte viability, attenuation of mitochondrial production of reactive oxygen species, and improved calcium handling, suggesting pleiotropic benefits of ivabradine treatment which are independent of HR reduction [23]. Recent data suggest that ivabradine treatment may attenuate post-ischaemic myocardial stunning [24, 25] and therefore may potentially prevent deterioration of left ventricular dysfunction due to the development of myocardial hibernation, which is thought to result from repeated episodes of myocardial stunning [26].

## Pharmacokinetics, Drug Interactions and Tolerability

**Fig. 2:**
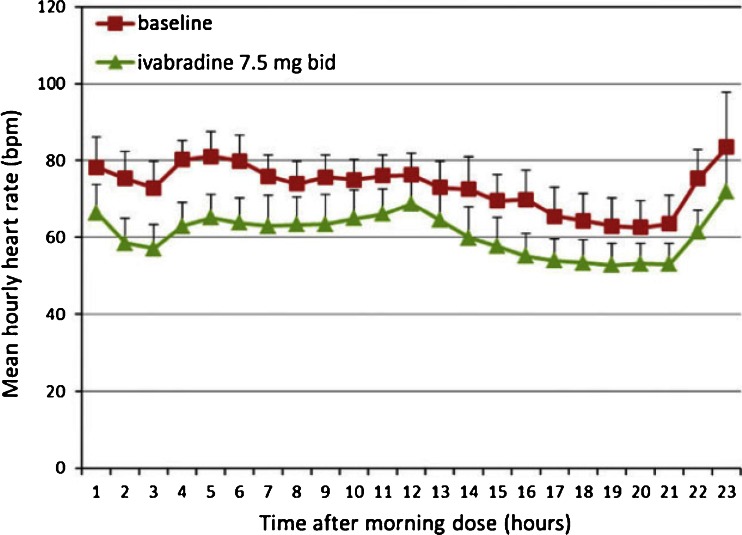
Change from baseline in mean heart rate over 24 h after treatment with ivabradine7.5 mg twice daily in volunteers. Bpm, beats per minute. Clinical data from the IRIS trial. EudraCT record 2011–001,665-40 (data on file). Reproduced from [65]

Ivabradine is rapidly absorbed after oral administration with linear pharmacokinetics in the dose range of 0.5–24 mg [27]. Under fasting conditions, the time to peak drug concentration is approximately 1 h, with an absolute bioavailability of film-coated tablets of ~40 % following both gastrointestinal and hepatic first-pass metabolism. By contrast, in fed conditions, the time to peak plasma drug concentration is prolonged by approximately 1 h with the plasma concentration being ~20–30 % higher. Approximately 70 % of ivabradine is plasma protein bound. Ivabradine has a half-life of 2 h in plasma and a biological half-life of 11 h, resulting in a significant reduction in HR with preservation of diurnal heart rate variation (Fig. 2). Approximately 4 % of a single oral ivabradine dose is excreted unchanged in urine. Renal impairment has minor impact on the pharmacokinetics of both ivabradine and its main metabolite, as renal clearance accounts for only ~20 % of the elimination of both products, though care should be taken in patients with advanced renal dysfunction. Patients with mild hepatic impairment require no dose adjustment. Ivabradine may be used with care in patients with moderate hepatic impairment, but is contraindicated in cases of severe hepatic insufficiency. No significant differences in the pharmacokinetic profile of ivabradine have been observed with age, though dosing should be judicious in patients aged >75 years.

Ivabradine is extensively metabolised by the cytochrome P450 (CYP) enzyme CYP3A4. It is a very weak inhibitor of this enzyme and does not appear to influence metabolism and plasma concentrations of other CYP3A4 substrates. By contrast, CYP3A4 inhibitors and agonists have been demonstrated to alter ivabradine plasma concentrations. The concomitant use of moderate or strong CYP3A4 inhibitors (e.g. non-dihydropyridine calcium antagonists, macrolide antibiotics, anti-retroviral drugs, anti-fungals) is contraindicated. Proton pump inhibitors, sildenafil, 3-hydroxy-3-methyl-glutaryl-CoA (HMG-CoA) reductase inhibitors, dihydropyridine calcium-channel blockers, digoxin and warfarin have not demonstrated any clinically significant effect on the pharmacokinetics and pharmacodynamics of ivabradine.

Ivabradine is generally well-tolerated in both clinical trials and real world observational studies, and described in greater detail below. Common minor side-effects are mainly visual (phosphenes, blurred vison and occasionally stroboscopic effects) and gastrointestinal. These are generally mild, transient and rarely require treatment discontinuation. The commonest cardiac side-effects are bradycardia (asymptomatic and symptomatic), atrial fibrillation and QT prolongation, though after correction for heart rate, QTc is only modestly increased and should not significantly increase risk of torsade de pointes [28]. Side effect profiles in the context of reported clinical trials will be discussed in greater detail below.

## Ivabradine as Monotherapy

The first randomised placebo-controlled trial of ivabradine in patients with stable angina was reported in 2003, in which ivabradine at doses of 5 and 10 mg twice daily was superior to placebo in reducing HR, frequency of angina episodes and the use of short-acting nitrates, while increasing exercise tolerance and time to onset of ischemia on treadmill exercise testing, in a dose-dependent fashion [29].

The anti-ischaemic and anti-anginal effects of ivabradine (7.5 mg BD and 10 mg BD) were compared with atenolol (100 mg OD) in the randomised double blind INITIATIVE trial. In this study, ivabradine monotherapy was demonstrated to be non-inferior to atenolol for increasing total exercise duration, time to limiting angina, time to angina onset and time to 1 mm ST depression. Patients treated with ivabradine reported decreased angina frequency and short acting nitrates (SAN) use similar to that observed in atenolol treated patients [30].

After 3 months, ivabradine (7.5 or 10 mg BD) was non-inferior to amlodipine (10 mg OD) for increasing total exercise duration, as well as the time to onset of ischaemia and angina on treadmill exercise testing. Both drugs produced a 60 % decrease in the frequency of angina attacks and a 50–60 % drop in the use of SAN. Compared to the dihydropyridine-calcium channel blocker, ivabradine lowered HR both at rest and at peak exercise with a consequent greater reduction of the rate-pressure product [31].

These data suggest that ivabradine monotherapy is equivalent to guideline recommended first-line drugs for improving angina symptoms and exercise testing parameters in patients with SCAD.

## Ivabradine as Add-On Therapy in Patients with SCAD

As discussed above, increased heart rate is a major determinant of myocardial ischemia [32] and has been a major target for pharmacologic intervention. Beta blockers are the exemplar negatively chronotropic drugs and are recommended as first-line therapy for angina in both European and US clinical guidelines. However, beta blockers at target dose are often poorly tolerated [33], which can result in suboptimal heart rate control and persistent anginal symptoms requiring the use of additional pharmacologic agents. There is only limited evidence that the addition of long-acting nitrates, nicorandil, or dihydropyridine calcium antagonists to beta blocker therapy results in improved symptoms, objective markers of myocardial ischemia, and exercise duration [34–38]. There is, therefore, a clinical need for pharmacologic strategies which can be safely added to beta blockade, that are well tolerated, provide objective reduction of ischaemic burden, improve exercise duration, and reduce anginal symptoms without an increased risk of adverse events. Furthermore, epidemiologic data also suggest a relationship between resting heart rate and prognosis (all-cause death, sudden cardiac death and fatal myocardial infarction) across the spectrum of cardiovascular disease [7, 39, 40]. In patients with coronary artery disease enrolled in the CASS study, resting HR was a potent and independent predictor of all-cause and cardiovascular mortality [40]. In previous studies of patients with systolic heart failure [41] or previous myocardial infarction [42], improved prognosis was correlated with the magnitude of heart rate reduction. Hypothetically, reducing heart rate has the potential not only to relieve symptoms of angina but also to improve prognosis in patients with SCAD.

**Fig. 3:**
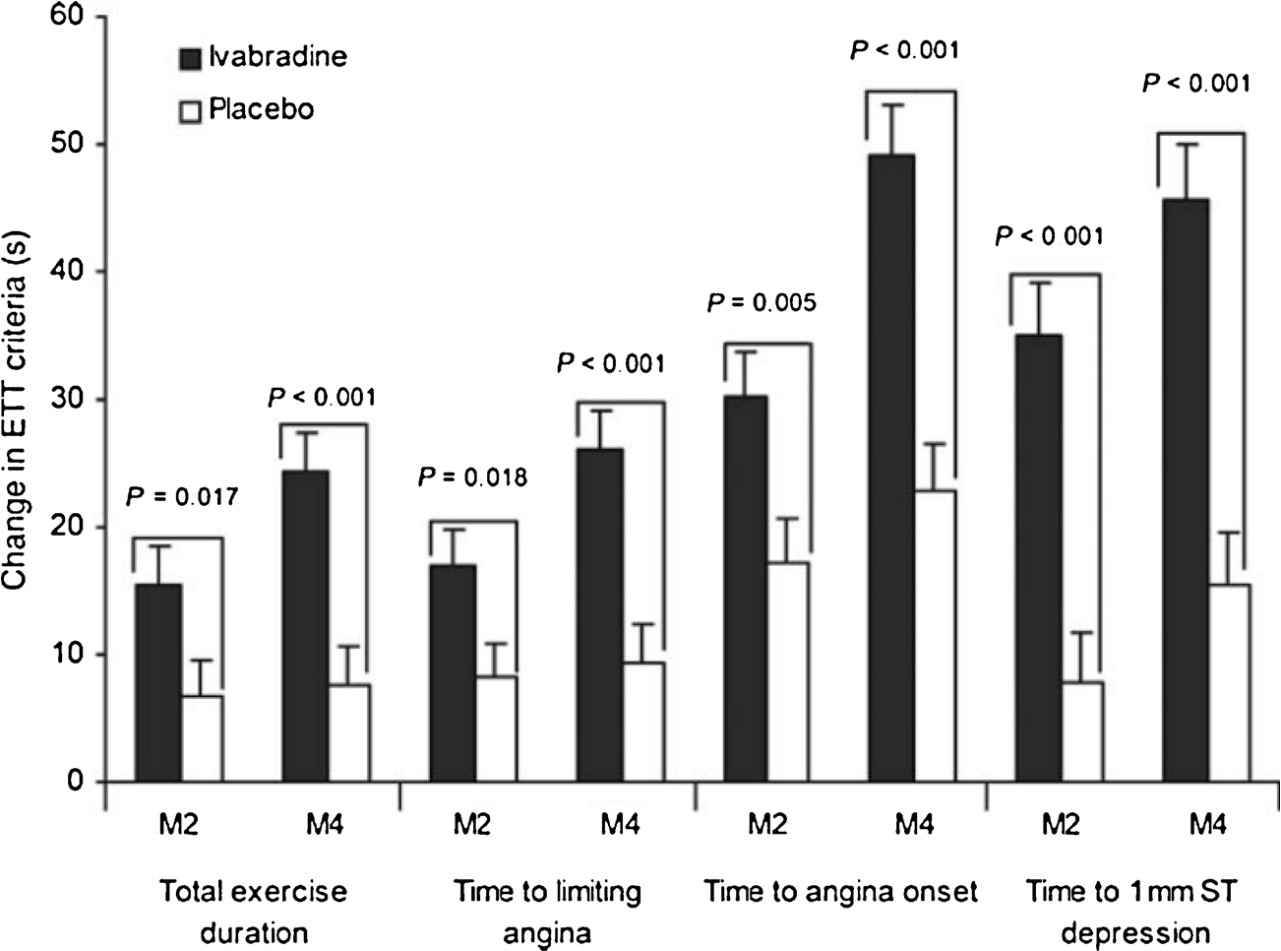
Changes in exercise tolerance test criteria between baseline and two month (M2) visit and between baseline and four month end of study (M4) in the full analysis set of the ASSOCIATE study. Reproduced from [44]

Previous attempts to optimise heart rate control by adding non-dihydropyridine calcium antagonists on top of background beta blocker therapy have been unsuccessful due to the development of limiting side-effects [43] and should therefore be avoided. More recently, in the ASSOCIATE trial, ivabradine (5 to 7.5 mg BD) as add-on therapy in patients already established on beta blockers was effective in significantly improving exercise duration and time to both electrocardiographic evidence of ischaemia and symptomatic angina on treadmill exercise testing (Fig. 3), though the frequency of self-reported anginal attacks was not significantly reduced [44, 45]. Serious or symptomatic bradycardia occurred in only 1.1 % of patients. These data provided the basis for randomised clinical trials evaluating the effect of heart rate reduction by ivabradine on clinical outcomes in patients with SCAD with both impaired and normal left ventricular function.

## Randomised Clinical Outcomes Trials of Ivabradine in SCAD

The BEAUTIFUL (morBidity-mortality EvAlUaTion of the I*f* inhibitor ivabradine in patients with coronary disease and left-ventricULar dysfunction) trial was an international multi-centre randomised double-blind placebo controlled trial that tested the hypothesis that reduction of heart rate with ivabradine (5 to 7.5 mg BD) in patients with SCAD and LVEF <40 % on established standard therapy would reduce both mortality and morbidity [46]. The primary endpoint was the composite of cardiovascular death, admission to hospital for acute myocardial infarction, and admission to hospital for new-onset or worsening heart failure. The study was designed with 90 % power to detect a 19 % difference in the primary endpoint based on an estimated event rate of rate of 11 % at 2.25 years in the placebo group. The observed event rate was significantly higher than predicted and the trial was modified such that all randomised patients were followed up for 12 months. Of 12,473 patients screened to enter the study, 10,917 eligible patients were randomised to receive either placebo (5438 patients) or ivabradine (5479 patients). Mean HR was 71.6 bpm with 87 % of patients receiving beta blockers at randomisation. Ivabradine therapy was well tolerated with symptomatic bradycardia occurring in ~5 % of patients. Ivabradine significantly reduced heart rate by ~6 bpm at 12 months compared to placebo. However, this did not translate into a significant improvement in the primary endpoint between ivabradine and placebo treated patients (15.4 % v 15.3 %, hazard ratio 1·00, 95 % CI 0·91–1·1, *p* = 0·94). In a pre-specified analysis of patients with HR >70 bpm at randomisation, there was no significant difference in the primary endpoint (hazard ratio 0·91, 95 % CI 0·81–1·04, *p* = 0·17) or the secondary endpoints of cardiovascular death or heart failure outcomes. However, the rates of non-fatal or fatal myocardial infarction (hazard ratio 0·64, 95 % CI 0·49–0·84, *p* = 0·001) and coronary revascularisation (hazard ratio 0·70, 95 % CI 0·52–0·93, *p* = 0·016) were significantly reduced. The authors proposed that these observed reductions in coronary events were consistent with the hypothesis that increased coronary atherogenesis [47] and risk of plaque rupture [48], driven by higher resting HR, may be potentially attenuated by ivabradine therapy.

Two major post-hoc analyses of the BEAUTIFUL trial have been reported. The first comprised an analysis of patients randomised to the placebo arm of the study to investigate the relationship between resting HR and clinical outcomes [49] and the second examined the effects of ivabradine therapy in those patients with limiting angina [50]. In the former study [49], the rates of cardiovascular death, hospitalisation for heart failure, hospitalisation for myocardial infarction and coronary revascularisation were all significantly higher in patients with resting HR >70 bpm, with an increase of 8 % (*p* = 0.0005), 16 % (*p* < 0.0001), 7 % (*p* = 0.052) and 8 % (*p* = 0.034) for every 5 bpm increment in HR in each of these endpoints, respectively. These results add to the body of data supporting the prognostic importance of increased heart rate, and in particular show a relationship between increased HR and adverse heart failure and coronary outcomes, in patients with SCAD and impaired LV function.

**Fig. 4:**
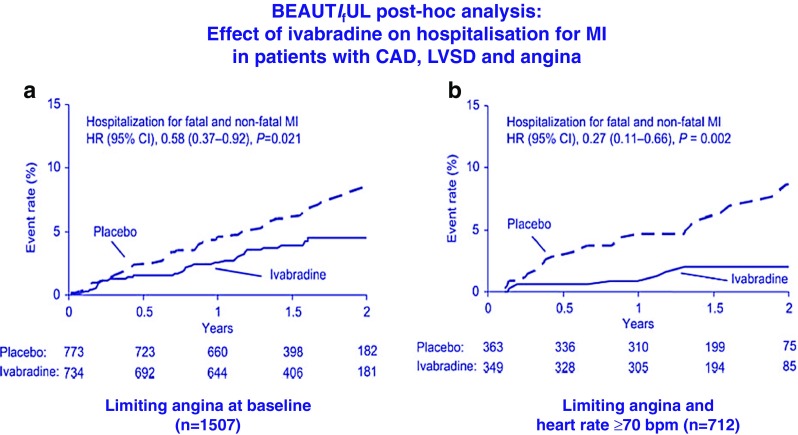
Post-hoc analysis demonstrating the effect of ivabradine treatment on hospitalisation for fatal and non-fatal myocardial infarction in the subgroup of patients in the BEAUTIFUL trial with symptomatic angina (**a**) and the subset with HR >70 bpm (**b**). Adapted from [50]

In an analysis of 1507 patients in the BEAUTIFUL trial population with limiting angina [50], ivabradine treatment was associated with a significant reduction in the trial primary endpoint (hazard ratio 0.76, 95 % CI 0.58–1.00, *p* = 0.05) and a significant reduction in hospitalisation for MI (hazard ratio 0.58, 95 % CI 0.37–0.92, *p* = 0.021) (Fig. 4). A further analysis stratified by HR >70 bpm, in addition, suggested a significant reduction in coronary revascularisation in ivabradine treated patients (hazard ratio 0.41, 95 % CI 0.17–0.99, *p* = 0.04). These data raised the hypothesis that ivabradine therapy may improve prognosis by reducing adverse coronary outcomes in patients with symptomatic SCAD, which has been tested recently [51].

The Study Assessing the Morbidity–Mortality Benefits of the *I*_f_ Inhibitor Ivabradine in Patients with Coronary Artery Disease (SIGNIFY) trial was an international multi-centre double-blind randomised placebo-controlled which recruited 19,102 patients in 51 countries with established SCAD, HR >70 bpm and no evidence of heart failure. Patients were randomised to receive ivabradine at an initial dose of 7.5 mg BD (except patients >75 years old who received a dose of 5 mg BD) or placebo. The ivabradine dose could be adjusted in the range 5-10 mg BD, to maintain a target HR of 55–60 bpm and to avoid symptomatic bradycardia. The composite primary endpoint consisted of death from any cause, cardiovascular death or non-fatal myocardial infarction. The trial was designed to provide 90 % power to detect an 18 % relative risk reduction with ivabradine, assuming an incidence of the primary outcome of 2.7 % per year.

**Fig. 5:**
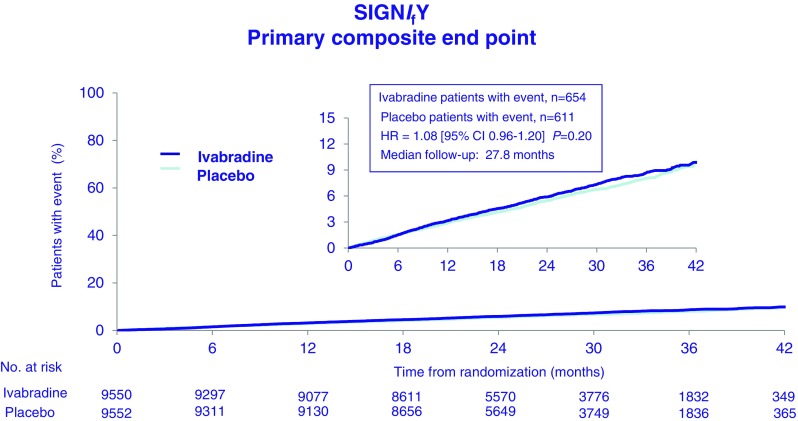
Primary composite endpoint (death from any cause, cardiovascular death or non-fatal myocardial infarction) in the SIGNIFY trial. Adapted from [51]

**Fig. 6:**
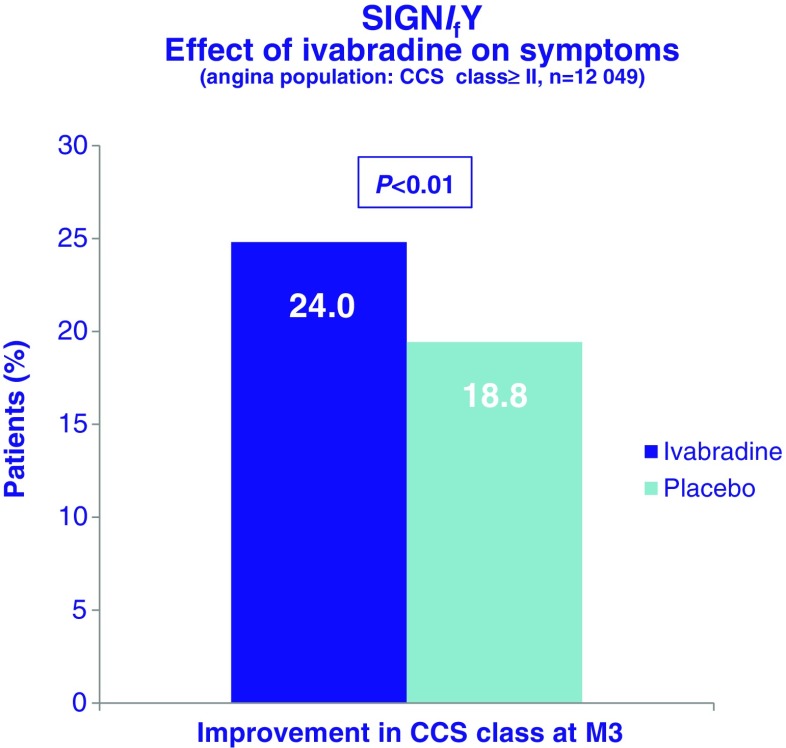
Effect of ivabradine on symptoms of angina as defined by CCS class in the SIGNIFY trial. Adapted from [51]

**Fig. 7:**
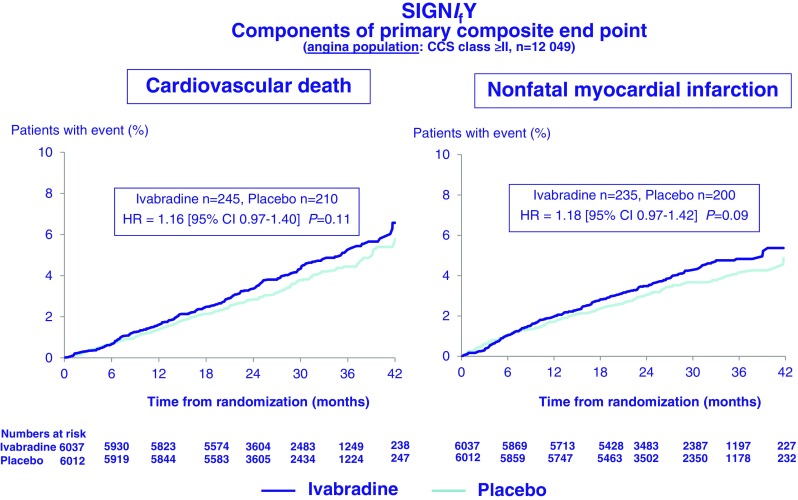
Components of the primary composite endpoint pre-specified limiting angina subgroup in the SIGNIFY trial. Adapted from [51]

**Fig. 8:**
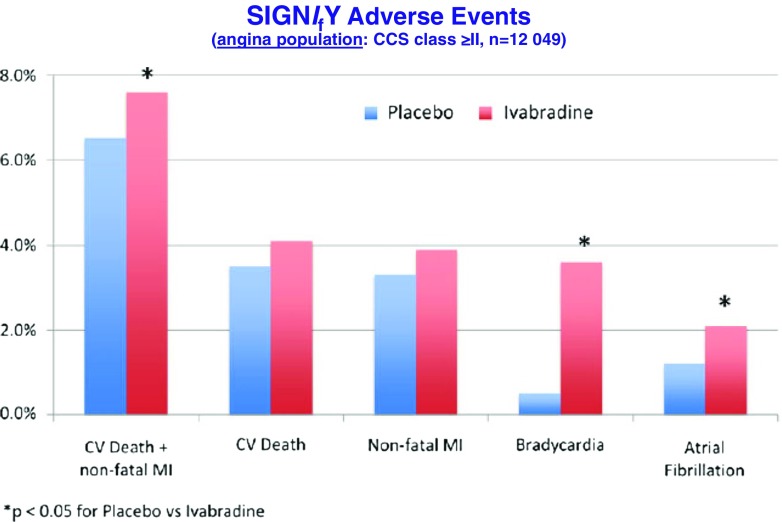
Analysis of adverse events in the subgroup of patients with moderate or severe angina (CCS class II-IV) in the SIGNIFY trial. CV, cardiovascular; MI, myocardial infarction. Adapted from [66]

The mean study-drug dose was 8.2 ± 1.7 mg BD in the ivabradine treatment arm. At 3 months, mean HR in the ivabradine treated group was 60.7 ± 9.0 bpm compared with 70.6 ± 10.1 bpm in the placebo arm, with this difference in HR being maintained for the duration of the study. Compliance with study medication was high. Change in beta blocker dose was infrequent in both study arms. Disappointingly, intention to treat analysis of the entire study cohort failed to demonstrate any difference in the primary composite endpoint (hazard ratio 1.08; 95 % CI 0.96–1.20, *p* = 0.20) (Fig. 5) or in any of the secondary endpoints, including non-fatal myocardial infarction, between the ivabradine and placebo groups. All-cause death, cardiovascular death and sudden death were also not significantly different in the two groups. Angina symptoms significantly improved in patients randomised to ivabradine (*p* = 0.01) (Fig. 6), and while subsequent analyses have reported better Seattle Angina Questionnaire scores, particularly in the area of disease perception, though the domain relating to physical limitation was not significantly impacted by ivabradine treatment [52]. In a pre-specified subgroup analysis of 12,049 patients with CCS class ≥2 angina, ivabradine therapy was associated with an increase in the primary endpoint (7.6 %, v 6.5 % with placebo; hazard ratio, 1.18; 95 % CI, 1.03–1.35; *P* = 0.02) with consistent observations for both cardiovascular death (hazard ratio 1.16; 95 % CI, 0.97 to 1.40; *P* = 0.11) and non-fatal myocardial infarction (hazard ratio 1.18, 95 % CI, 0.97 to 1.42; *P* = 0.09) (Fig. 7). There was no interaction between the use of ivabradine and adverse events in other pre-specified sub-groups, defined according to age, beta blocker use at randomisation, gender, baseline HR, history of diabetes mellitus, previous MI, or previous coronary revascularisation. Ivabradine treatment, compared with placebo, was associated with significantly increased rates of symptomatic (7.9 % v 1.2 %, *p* < 0.001) and asymptomatic (11.0 % v 1.3 %, *p* < 0.001)) bradycardia, atrial fibrillation (5.3 % v 3.8 %, *p* < 0.001) and phosphenes (5.4 % v 0.5 %, *p* < 0.001). Study drug withdrawal occurred in 13.2 % of ivabradine treated patients compared to 7.4 % in the placebo group (*p* < 0.001). There was an absolute 2.2 % increase in serious adverse events in the ivabradine treated group (*p* = 0.001). Interestingly, QT interval prolongation was noted more frequently in ivabradine treated patients (Fig. 8).

These data confirm that ivabradine significantly improves angina in patients with SCAD but do not support the hypothesis that this drug, which purely reduces heart rate, improves prognosis in patients with SCAD. These results suggest that HR should be considered as a biomarker of future cardiovascular risk rather than a modifiable risk factor in patients with SCAD. We will discuss further the implications of the SIGNIFY results on the safe clinical use of ivabradine.

## Real World Clinical Experience of Ivabradine in Patients with SCAD

The open-label ADDITIONS trial evaluated the effect of add-on ivabradine in patients who remained symptomatic with angina despite background beta blocker therapy in routine clinical practice [53]. In this study, ~70 % of patients had CCS class 2 or 3 angina with 24 % of patients treated with beta blockers at the target dose and 78 % were on at least half the target dose. The reduction in heart rate of 19.4 ± 11.4 bpm (*p* < 0.0001) observed following initiation of ivabradine was accompanied by significantly reduced SAN use, improved CCS class and minimal adverse events. Quality of life, as evaluated by EQ-5D index, significantly improved by 0.17 ± 0.23 (*p* < 0.0001) and 97 % of physicians rated the addition of ivabradine as having “good” or “very good” clinical efficacy. Additional studies have suggested the safety and clinical efficacy of ivabradine in patients in whom administration of beta blockers can be particularly challenging, including diabetics, the elderly, peripheral artery disease, chronic obstructive airways disease and asthmatics [1, 54–57]. Finally, preliminary data suggest that ivabradine may be considered to improve symptoms and quality of life in patients with microvascular angina [58], though only anecdotal evidence is currently available that shows benefit in patients with refractory angina.

## Should We Use Ivabradine for Management of Angina Post-SIGNIFY?

The results of SIGNIFY are perplexing – patients with symptomatic angina who are likely to benefit most from ivabradine therapy simultaneously appear to be those at greatest risk of developing adverse events. While the precise explanations for these observations remain unknown, four issues warrant further consideration, namely the use of an unlicensed drug dose, drug interactions, arrhythmia, and statistical error.

Firstly, the dosing strategy employed in the SIGNIFY trial, significantly differs from current clinical practice, where ivabradine is usually initiated at 2.5–5.0 mg BD and titrated to a maximum licensed dose of 7.5 mg BD. Forty-seven percent of ivabradine treated patients <75 years of age received ivabradine at a dose of 10 mg BD. Importantly, in the pre-specified subgroup of patients with limiting angina, the primary endpoint occurred at the 10 mg BD dosage in 58 % of patients. In a nested case control analysis, bradycardia was more common in patients exposed to a 10 mg BD dose of ivabradine, with emergent bradycardia being less frequent if ivabradine was initiated at a dose of 5 mg BD. Secondly, in the SIGNIFY trial, 4.6 % of patients were treated with verapamil or diltiazem, which are moderate inhibitors of CYP3A4, resulting in ~3-fold increases in plasma ivabradine levels and a pharmacodynamic interaction resulting in augmented negative chronotropic effects [59]. Concurrent use of verapamil or diltiazem was associated with a 68 % increase in the primary endpoint (*p* = 0.088) and an 88 % increase in non-fatal myocardial infarction (*p* = 0.026). These data underscore the importance of not co-prescribing verapamil or diltiazem with ivabradine and underpin recent recommendations from the Pharmacovigilance Risk Assessment Committee of the European Medicines Agency [59]. Thirdly, there may be potentially important pro-arrhythmic effects of ivabradine treatment [60]. While symptomatic bradycardia was more common in ivabradine treated patients, this was not found sufficient to account for the increased rate of adverse events in either the entire study cohort or the subgroup with limiting angina [60]. Emergent atrial fibrillation occurred in 754 patients and occurred more commonly in ivabradine treated patients. These data are consistent with recent meta-analyses which also suggest that those with higher pre-treatment resting HR may be at increased risk [13]. The presence of the HCN4 isoform of the *I*_f_ channel in myocyte sleeves surrounding the pulmonary veins may be an important aetiological factor [14]. Notably, the rate of the primary endpoint was not significantly increased in patients with emergent AF in either the whole study cohort or the subgroup with limiting angina [60]. Ivabradine treatment was associated with prolongation of the QT interval. Importantly, this was not associated with a significant rate of drug withdrawal or ventricular tachyarrhythmia. That said, ivabradine is contraindicated in patients with long QT syndromes and has been associated with an increased predisposition to torsade de pointes [61–63]. Finally, it should be emphasised that the results of subgroup analyses from trials that fail to meet the primary endpoint should be interpreted with caution due to the possibility of statistical error.

Over the last fifteen years, the anti-anginal efficacy of ivabradine has been evaluated in a clinical trial program that has recruited in excess of 35,000 patients with SCAD and symptomatic angina, with or without left ventricular dysfunction, either as monotherapy or in addition to first line anti-anginal drugs. That ivabradine improves symptoms but not prognosis in patients with SCAD and preserved LV function, does not place ivabradine in a unique position amongst drugs which are currently recommended for the treatment of symptomatic angina in clinical guidelines. There are no definitive randomised clinical trial data which demonstrate the prognostic benefits of any of the anti-anginal drugs, including beta blockers [64]. The available data suggest that ivabradine is a well-tolerated and effective anti-anginal agent. As recommended by the Pharmacovigilance Risk Assessment Committee of the European Medicines Agency [59], following their scrutiny of the SIGNIFY trial data, ivabradine should be used in patients with HR >70 bpm who are intolerant of or remain symptomatic despite the use of first line anti-anginal drugs. The dose of ivabradine should be up-titrated according to patient’s symptoms rather than aiming for a specific target HR [59]. The prescribed dose should not exceed 7.5 mg BD. Co-prescription of ivabradine with moderate or severe inhibitors of CYP3A4, in particular non-dihydropyridine calcium antagonists, is contraindicated. Patients should be carefully monitored for the development of bradycardia and atrial fibrillation, and ivabradine treatment should be avoided in patients at risk of torsade de pointes.

Ivabradine remains licensed for the treatment of symptomatic angina following review by the Pharmacovigilance Risk Assessment Committee of the European Medicines Agency [59]. Considerable further work will be needed to understand fully the observations from the SIGNIFY trial. However, important insights have been gained into the safe and appropriate use of ivabradine, which should still be considered a useful therapeutic option to relieve symptoms and improve quality of life in suitably selected patients with SCAD.
